# Changing Epidemiology of Invasive Candidiasis in Children during a 10-Year Period

**DOI:** 10.3390/jof5010019

**Published:** 2019-02-22

**Authors:** Maria Noni, Angeliki Stathi, Ilia Vaki, Aristea Velegraki, Levantia Zachariadou, Athanasios Michos

**Affiliations:** 1 First Department of Pediatrics, Division of Infectious Diseases, “Aghia Sophia” Children’s Hospital, National and Kapodistrian University of Athens, 115 27 Athens, Greece; marianoni21@gmail.com (M.N.); ilia.vaki@yahoo.gr (I.V.); 2 Department of Microbiology, “Aghia Sophia” Children’s Hospital, 115 27 Athens, Greece; angelikiestathi@gmail.com (A.S.); levantia@otenet.gr (L.Z.); 3 Department of Microbiology, School of Medicine, National and Kapodistrian University of Athens, 115 27 Athens, Greece; aveleg@med.uoa.gr

**Keywords:** candidiasis, invasive, antifungal, children, resistance

## Abstract

*Candida* species are a common cause of invasive infection in neonates and children. The aim of our study was to evaluate the epidemiology and microbiology of invasive candidiasis (IC) in the largest tertiary Greek pediatric hospital during a 10-year period. A retrospective cohort study was performed from January 2008 to December 2017. Identification of species and antifungal susceptibility testing was performed according to the Clinical and Laboratory Standards Institute (CLSI) methodology. During the study period, 178 cases of IC were recorded. The tissue distribution included blood (87.1%), cerebrospinal (7.9%), peritoneal (3.9%) and pleural fluids (1.1%). *Candida albicans* and *Candida parapsilosis* (*sensu lato*) were the most frequently isolated species (47.8% and 28.7% respectively). From period 2008–2012 to period 2013–2017, a significant decrease in IC rates was detected (0.21 cases/1000 hospitalization days VS 0.11 cases/1000 hospitalization days, *P* = 0.040), while median minimum inhibitory concentrations (MICs) of amphotericin B were significantly increased for both *C. albicans* and *C. parapsilosis* (*sl*) (*P* = 0.037 and *P* = 0.004 respectively). The decrease in IC rates may reflect the increased awareness as well as the effective infection control initiatives and antifungal interventions. However, the significant increase in the MICs for amphotericin B and echinocandins such as caspofungin, raises concerns about their common use as first-line treatment. Epidemiologic monitoring is, therefore, critically important in order to evaluate and optimize therapeutic protocols for IC in pediatric populations.

## 1. Introduction

Invasive candidiasis (IC) is considered as a major cause of mortality in neonates and children [1]. Today, several factors are considered as unique risk factors for candidemia in pediatric population such as prematurity and intensive care unit admission in neonates as well as neutropenia and hematologic malignancy in children [2]. Research areas in epidemiology, pharmacokinetics and diagnostics still have important knowledge gaps to fill in and as a result, consensus guidelines for IC in pediatric populations lack evidence-based recommendations [3,4].

Despite the fact that *C. albicans* is still considered the most commonly isolated species in IC, the frequency of non-*albicans Candida* (NAC) has increased during the last years [5]. Geographical differences in epidemiology trends as well as changes in resistance rates have also been detected [6,7]. Resistance to fluconazole varies significantly among countries, while the increased use of echinocandins seems to affect the emergence of resistance in Candida species [8]. These observations highlight the need of a frequent update of the local epidemiology data.

As limited data are available for IC in the Greek pediatric population, our aim was to investigate the epidemiology and microbiology of invasive candidiasis in the largest tertiary Greek pediatric hospital during a 10-year period.

## 2. Materials and Methods

### 2.1. Study Design

A retrospective cohort study of invasive infections caused by *Candida* species was performed at “Aghia Sophia” Children’s Hospital in Athens. This is a 750-bed tertiary hospital that is a referral center for almost 75% of the Greek pediatric population, and includes 3 neonatal intensive care units, 1 pediatric ICU, 2 hematology-oncology units and 1 bone marrow transplantation unit. All pediatric patients 18 years of age or younger who received treatment for invasive candidiasis from January 2008 to December 2017 were included. Invasive candidiasis was defined according to the European Organization for Research and Treatment of Cancer/Invasive Fungal Infections Cooperative Group and the National Institute of Allergy and Infectious Diseases Mycoses Study Group (EORTC/MSG) Consensus Group (EORTC/MSG) consensus revised definitions [9]. Briefly, proven disease required mycological documentation from a normally sterile site, whereas probable disease required a host factor, clinical and mycological evidence. Date of infection was defined as the date of clinical disease connected to the first positive culture for Candida that met disease criteria [9]. If multiple episodes of invasive candidiasis occurred in a single patient, episodes separated by clinical and microbiological resolution (defined by ≥14 days) were treated as a new episode.

### 2.2. Mycology and Antifungal Susceptibility Test

Blood, peritoneal and pleural fluid specimens were cultured in the BD BACTEC^TM^ automated system (BD Diagnostics) while celebrospinal fluid (CSF) directly on Sabouraud Dextrose Agar (SDA) (Bioprepare®-Microbiology, Athens, Greece) and in Sabouraud Dextrose Broth (SDB) (homemade). Yeast pathogens from positive cultures were sub-cultured onto SDA and Candida Chrom Agar (CCA) (Bioprepare®), processed for identification by micromorphology and carbohydrate assimilation testing (API® 32 °C, (BioMérieux, Marcy l’Etoile, France), enzymatic RapID Yeast-Plus System (Remel ThermoFisher Scientific^TM^ Lexena, KS) and Vitek2 (BioMerieux, Marcy l’Etoile, France) and frozen at −70 °C.

For susceptibility testing, isolates were freshly sub-cultured and analysed; Regarding *C. albicans* Clinical Break Points (CBP) were used for fluconazole, itraconazole, voriconazole and caspofungin and epidemiological cutoff values (ECV) were used for amphotericin B, flucytosine and posaconazole according to the Clinical and Laboratory Standards Institute (CLSI) protocol [10]. Regarding *C. parapsilosis,* CBP were used for fluconazole, voriconazole and caspofungin and ECV were used for amphotericin B, flucytosine, itraconazole and posaconazole. MIC values were measured for amphotericin B, flucytosine, fluconazole, itraconazole, posaconazole, voriconazole, and caspofungin by gradient concentration of the antifungal drug in strips applied on inoculated 0.5 McFarland yeast suspension turbidity 90mm-diameter RPMI agar plates with MOPS in a 1.5% agar base, supplemented with 2% glucose (Liofilchem s.r.l®, Roseto degli Abruzzi, Italy) and incubation at 35 °C for 24–48 h. MICs were read at the intersection point of the growth with concentration scale, taking into consideration the “trailing” effect where was appropriate. Antifungal susceptibility rates were calculated based on the CLSI breakpoints for all *Candida* species except in the case of the *Candida rugosa*, where the antifungal susceptibility was determined on the basis of bibliographic data [10,11].

### 2.3. Statistical Analysis

Descriptive analysis was performed for all variables. Categorical data were expressed as absolute number and proportions (%), while continuous variables were reported as the median and interquartile range (IQR), in the case of non-normal (Gaussian) distribution. Continuous data were tested for normality using statistical tests (Kolmogorov–Smirnoff test) and graphical methods (histogram, Q–Q plot). For normally distributed variables, Student’s *t*-test was used to assess differences between two groups, whereas for skewed variables, the Mann–Whitney U-test was performed. The rate of IC was calculated as the ratio between number of IC and days of hospitalization for each year in study and expressed as episodes/1000 hospitalization days. Coefficient of variation was calculated for rates of IC caused by *C. albicans* and NAC during the study period. Levene’s test was performed to compare the differences in coefficient of variations. All statistical analyses were performed with the statistical package PSAW Statistics v23 (SPSS, Inc., Chicago, IL, USA). Statistical significance at *P* < 0.05 was assumed.

### 2.4. Ethical Approval

The study was reviewed and approved (5878/07-03-2014) by The Hospital’s Research Ethics Board.

### 2.5. Informed Consent

Not applicable. This is a retrospective cohort study over a prolonged period of time and complete anonymity was achieved.

## 3. Results

During the 10-year period, 178 cases of invasive candidiasis (IC) were recorded, and ten different *Candida* species were detected (Table 1). *C. albicans* was the most frequently isolated species (*n* = 85, 47.8%). *C. parapsilosis* (*sl*) was the most commonly isolated NAC (*n* = 51, 28.7%) followed by *Candida lusitaniae* (*n* = 11, 6.2%), *Candida tropicalis* (*n* = 8, 4.5%)*, Candida glabrata* (*n* = 8, 4.5%), *Candida famata* (*n* = 6, 3.4%) *Candida krusei* (*n* = 5, 2.8%), *Candida guilliermondii* (*n* = 2, 1.1%), *Candida kefyr* (*n* = 1, 0.6%) and *Candida rugosa* (*n* = 1, 0.6%).

*Candida* species were isolated from peripheral blood samples of 155 (87.1%) patients, CSF samples of 14 (7.9%) patients, peritoneal fluid samples of 7 (3.9%) patients and pleural fluid samples of 2 (1.1%) patients. In all samples, the most commonly isolated agent was *C. albicans*. Regarding NAC, *C. parapsilosis* (*sl*) was the species with the highest frequency (Table 1).

Seventy-seven cases (43.3%) were detected in the Neonatal Intensive Care Unit (NICU), 23 (12.9%) in Pediatric Units (PU), 26 (14.6%) in the Hematology/Oncology Unit (HOU), 37 (20.8%) in the Pediatric Intensive Care Unit (PICU) and 15 (8.4%) in the Surgical Unit (SU). When the units were examined by the distribution of species, the most commonly isolated agent was again *C. albicans*, followed by *C. parapsilosis* (*sl*)*. C. albicans* was the most frequently isolated species in PICU (*n* = 20, 54.1%), NICU (*n* = 40, 51.9%), HOU (*n* = 9, 34.6%) and SU (*n* = 8, 53.3%), while *C. parapsilosis* (*sl*) was the most commonly isolated species in PU (*n* = 9, 39.1%). Regarding the other non-common NAC species, such as *C. lusitaniae*, *C. tropicalis* and *C. glabrata*, the majority of them were detected in PICU and NICU. Differences that were observed between clinics regarding *C. albicans* and NAC isolations were not statistically significant (*P* = 0.323). Moreover, differences that were observed between ICU (PICU and NICU) and non-ICU (PU, SU and HOU) regarding *C. albicans* and NAC were also statistically non-significant (*P* = 0.082) (Table 2).

Table 3 summarizes data regarding IC rates per 1000 hospitalizations in different pediatric units. According to our data, the highest mean IC rate during the 10-year period was observed in PICU (0.60 ± 0.45), followed by NICU, HOU and PU (0.15 ± 0.08 and 0.15 ± 0.13, respectively). Statistical analysis revealed a significant decrease in the IC rate from the first to the second five-year period. In detail, the incidence decreased from 0.21 cases/1000 hospitalization days between 2008 and 2012 to 0.11 cases/1000 hospitalization days to 2013 and 2017 (*P* = 0.040). The same trend was observed in all hospital units but differences were not statistically significant.

During the study period, the coefficient of variation for IC rates per 1000 hospitalization days caused by *C. albicans* was 0.587, while for IC rates caused by NAC, 0.473. This difference was not statistically significant when Levene’s test was performed (*F* = 1.63, *P* = 0.201).

Susceptibility rates of *C. albicans* to the selected antifungal drugs are shown in Table 4. Non-wild type (NWT) for amphotericin B was found in 1.2% of isolates, while for flucytosine in 4.8% and for posaconazole in 37.6%.

Among *C. albicans* isolates, fluconazole resistance was not detected (0.0%), whereas for itraconazole, this was 4.7% and for voriconazole 0.0%. Regarding caspofungin, 97.7% of isolates were found to be susceptible, while none of isolates was considered resistant.

When median MICs were compared among five-year periods, a significant increase was detected regarding amphotericin B and caspofungin (*P* = 0.037 and *P* = 0.019, respectively). No significant differences were noticed in median MICs among the two periods for the rest of the antifungal drugs (Table 5).

Susceptibility rates of *C. parapsilosis* (*sl*) to the selected antifungal drugs are shown in Table 4. NWT for amphotericin B was found in 2.0%, while for flucytosine, in 5.9%, for itraconazole, in 8.0% and for posaconazole in 15.4%. The resistance in fluconazole was 3.9% among *C. parapsilosis* (*sl*) isolates, whereas in voriconazole, this was 0.0%. Regarding caspofungin, 98.1% of isolates were found to be susceptible, while none of isolates was considered resistant. During the second five-year period, a significant increase was detected in median MICs regarding amphotericin B (*P* = 0.004). An increase in median MICs was also detected for caspofungin but was not statistically significant (*P* = 0.279). No significant differences were noticed in median MICs for the rest antifungal drugs (Table 5).

## 4. Discussion

In the present study, we describe the epidemiology of invasive candidiasis in neonatal and pediatric populations in the largest tertiary Greek pediatric hospital during a 10-year period. According to these results, a significant decrease was observed during the last five years in IC rates. In addition, it was shown that median MICs of amphotericin B were significantly increased for both *C. albicans* and *C. parapsilosis* (*sl*) as well as median MICs of caspofungin for *C. albicans*. Although, changes over time should be interpreted with caution especially when they are small, they reflect the trends of antifungal resistance in our pediatric population. Another quite important result was the fact that no resistance to fluconazole was detected for *C. albicans* isolates through the study period.

The average incidence of candidemia during the 10-year period was approximately 0.158 per 1000 hospitalization days (range from 0.02 to 0.26 per hospitalization days). These rates were among the lowest of those reported from surveys conducted in some European countries as well as in the USA [12,13,14,15]. In addition, the significant decrease in IC rates that was observed was consistent with other long-term pediatric studies [14]. Multiple hypotheses might explain this clinically significant decline. The increased awareness of IC, effective infection control initiatives and improved therapeutic as well as prophylactic antifungal interventions could have contributed decisively to this result and should be studied further.

Throughout the study period, *C. albicans* was the most frequently isolated *Candida* species, but NAC species predominated overall. This trend is in line with the world-wide shift from *C. albicans* to NAC that has been noted since the 1990s in the species responsible for candidemia [16]. Statistical assessment revealed no difference between the clinical units in terms of *C. albicans* and NAC, but the frequency of NAC was high in HOU as well as PU. Regarding NU, although no difference was detected between *C. albicans* and NAC, *C. albicans* predominated over *C. parapsilosis* (*sl*). Similar isolation rates among units were reported from a Turkish study that was published recently. However, *C. albicans* and NAC were equally distributed in the Turkish clinic [17]. In PICU, NAC was isolated in 45.9% of cases, a percentage that is 8.6% lower than the one reported by Vogiatzi et al in a 5-year retrospective multicenter Greek study [18]. Our observations highlight the importance of separately analyzing pediatric and neonatal patients in future studies, as well as closely monitoring local species distribution.

Recent guidelines for the management of IC in both neonates and children favor the use of echinocandins, fluconazole and amphotericin B [3,4]. Echinocandins are first-line agents for the treatment of IC in children. Regarding neonates, there is still limited clinical and safety data, although their use has increased among NICUs [19]. The significant increase in MICs of caspofungin for *C. albicans* that was detected during the last five years enhances general worries about increasing rates of resistant species [20,21]. According to some studies, caspofungin susceptibility could sometimes predict anidulafungin and micafungin susceptibility [22]. However, taking into consideration that resistance may develop with different mechanisms, our results highlights the need to specify the susceptibility to each echinocandin separately. On the other hand, the already published cases of misclassification of susceptible isolates of *C. glabrata* and *C. krusei* associated with the revised CLSI caspofungin breakpoints enhance the concerns about the validity of strips against micro-dilutions as well as the need of further necessary studies comparing the two methods [23]. Fluconazole has been recommended as the best alternative drug for patients with IC due to its safety and efficacy [3,4]. Although our data revealed the absence of resistance to fluconazole in *C. albicans*, a remarkable percentage of *C. parapsilosis* (*sl*) resistant to fluconazole was detected (3.9%), which was quite the same with those reported in Italian studies [24,25]. Although MICs rates did not increase significantly during the study period, these findings may act as a barrier against fluconazole prophylaxis and empirical use in the future [26].

Amphotericin B is an alternative therapeutic option when isolates exhibit resistance against azoles or echinocandins [3,4]. The increased MICs of amphotericin B for both *C. albicans* and *C. parapsilosis* (*sl*) found in our study raise serious concerns, as resistance to amphotericin B has been rarely reported worldwide [17,24,27].

The present study has some potential limitations. Although it is a single-center study, it is the largest reference center in our area, offers laboratory uniformity and the results are comparable and standardized. However, in order to have larger isolate numbers, multicenter studies are needed.

In conclusion, our results indicate a significant decrease in IC through the last decade. The etiology of this decline is likely multifactorial and, thus, further studies are necessary to clarify possible factors. However, the increase in MICs for specific antifungals that was detected is quite worrisome and highlights the importance of monitoring and surveillance in order to optimize antifungal therapeutic guidelines.

## Figures and Tables

**Table 1:** Distribution of *Candida* species by infection site during the study period (2008–2017).

*Candida* Species (*n* = 178)	Blood (*n* = 155)	CSF (*n* = 14)	Peritoneal Fluid (*n* = 7)	Pleural Fluid (*n* = 2)
***C. albicans* (*n* = 85)**	74 (47.7)	7 (50.0)	3 (42.9)	1 (50.0)
***C. parapsilosis* (*sl*) (*n* = 51)**	45 (29.0)	3 (21.4)	3 (42.9)	0 (0.0)
***C. lusitaniae* (*n* = 11)**	9 (5.8)	2 (14.3)	0 (0.0)	0 (0.0)
***C. tropicalis* (*n* = 8)**	6 (3.9)	1 (7.1)	0 (0.0)	1 (50.0)
***C. glabrata* (*n* = 8)**	8 (5.2)	0 (0.0)	0 (0.0)	0 (0.0)
***C. famata* (*n* = 6)**	5 (3.2)	1 (7.1)	0 (0.0)	0 (0.0)
***C. krusei* (*n* = 5)**	4 (2.6)	0 (0.0)	1 (14.3)	0 (0.0)
***C. guilliermondii* (*n* = 2)**	2 (1.3)	0 (0.0)	0 (0.0)	0 (0.0)
***C. kefyr* (*n* = 1)**	1 (0.6)	0 (0.0)	0 (0.0)	0 (0.0)
***C. rugosa* (*n* = 1)**	1 (0.6)	0 (0.0)	0 (0.0)	0 (0.0)

CSF: Cerebrospinal fluid.

**Table 2:** Distribution of *Candida* species by units during the study period (2008–2017).

*Candida* Species (*n* = 178)	PICU (*n* = 37)	NICU (*n* = 77)	HOU (*n* = 26)	PU (*n* = 23)	SU (*n* = 15)	*p*-Value
***C. albicans* (*n* = 85)**	20 (54.1)	40 (51.9)	9 (34.6)	8 (34.8)	8 (53.3)	0.323
***Candida* non-*albicans* (*n* = 93)**	17 (45.9)	37 (48.1)	17 (65.4)	15 (65.2)	7 (46.7)	
***C. parapsilosis* (*sl*) (*n* = 51)**	6 (16.2)	24 (31.2)	8 (30.8)	9 (39.1)	4 (26.7)	
***C. lusitaniae* (*n* = 11)**	2 (5.4)	5 (6.5)	2 (7.7)	1 (4.3)	1 (6.7)	
***C. tropicalis* (*n* = 8)**	5 (13.5)	0 (0.0)	1 (3.8)	2 (8.7)	0 (0.0)	
***C. glabrata* (*n* = 8)**	0 (0.0)	7 (9.1)	0 (0.0)	0 (0.0)	1 (6.7)	
***C. famata* (*n* = 6)**	2 (5.7)	1 (1.3)	2 (7.7)	1 (4.3)	0 (0.0)	
***C. krusei* (*n* = 5)**	1 (2.7)	0 (0.0)	2 (7.7)	2 (8.7)	0 (0.0)	
***C. guilliermondii* (*n* = 2)**	1 (2.7)	0 (0.0)	1 (3.8)	0 (0.0)	0 (0.0)	
***C. kefyr* (*n* = 1)**	0 (0.0)	0 (0.0)	1 (3.8)	0 (0.0)	0 (0.0)	
***C. rugosa* (*n* = 1)**	0 (0.0)	0 (0.0)	0 (0.0)	0 (0.0)	1 (6.7)	

PICU: Pediatric Intensive Care Unit; NICU: Neonatal Intensive Care Unit; HOU: Hematology/Oncology Unit; PU: Pediatric Unit; SU: Surgical Unit.

**Table 3:** Comparison of invasive candidiasis rates per 1000 hospitalization days during the study period (2008–2017).

	2008–2017	2008–2012	2013–2017	*p*-Value
**Total invasive candidiasis/1000 hospitalization days**	0.16 ± 0.08	0.21 ± 0.06	0.11 ± 0.06	0.04
**PICU**	0.60 ± 0.45	0.72 ± 0.56	0.48 ± 0.31	0.43
**NICU**	0.15 ± 0.08	0.17 ± 0.09	0.14 ± 0.10	0.67
**HOU**	0.15 ± 0.13	0.17 ± 0.17	0.13 ± 0.08	0.65
**PU**	0.05 ± 0.03	0.06 ± 0.03	0.04 ± 0.04	0.41
**SU**	0.09 ± 0.06	0.11 ± 0.07	0.07 ± 0.05	0.32

Data are presented as the mean ± standard deviation (SD). PICU: Pediatric Intensive Care Unit; NICU: Neonatal Intensive Care Unit; HOU: Hematology/Oncology Unit; PU: Pediatric Unit; SU: Surgical Unit.

**Table 4:** Susceptibility rates of *C. albicans* isolates (*n* = 85) and *C. parapsilosis* (*sl*) isolates (*n* = 51) according to Clinical and Laboratory Standards Institute (CLSI) breakpoints during the study period (2008–2017).

Antifungal Agent	ECV (μg/mL)/(%)					CBP (μg/mL)/(%)							
	WT	(%)	Non-WT	(%)		S	(%)	SDD	(%)	I	(%)	R	(%)
***C. albicans***													
**Amphotericin B**	≤2	98.8	>2	1.2									
**Flucytosine**	≤0.5	95.2	>0.5	4.8									
**Fluconazole**	≤0.5		>0.5			≤2	98.8	4	1.2			≥8	0.0
**Itraconazole**	≤0.12		>0.12			≤0.12	84.7	0.25–0.5	10.6			≥1	4.7
**Posaconazole**	≤0.06	62.4	>0.06	37.6									
**Voriconazole**	≤0.03		>0.03			≤0.12	100.0			0.25–0.5	0.0	≥1	0.0
**Caspofungin**	≤0.12		>0.12			≤0.25	97.7			0.5	2.3	≥1	0.0
***C. parapsilosis***													
**Amphotericin B**	≤2	98.2	>2	1.8									
**Flucytosine**	≤0.5	94.1	>0.5	5.9									
**Fluconazole**	≤2		>2			≤2	92.2	4	3.9			≥8	3.9
**Itraconazole**	≤0.5	92.0	>0.5	8.0									
**Posaconazole**	≤0.25	84.6	>0.25	15.4									
**Voriconazole**	≤0.12		>0.12			≤0.12	98.1			0.25–0.5	1.9	≥1	0.0
**Caspofungin**	≤1		>1			≤2	98.1			4	1.9	≥8	0.0

ECVs, epidemiological cutoff values; CBPs, clinical breakpoints; WT, wild type; non-WT, non-wild type; S, susceptible; SDD, susceptible dose dependent; I, intermediate; R, resistant.

**Table 5:** Differences in antifungal MICs for *C. albicans* and *C. parapsilosis* (*sl*) between two 5-year periods (2008–2012 and 2013–2017).

	2008–2012 MIC (μg/mL)	2013–2017 MIC (μg/mL)	*p*-Value
***C. albicans***			
**Amphotericin B**	0.250 [0.094 0.500]	0.380 [0.220 0.500]	0.037
**Flucytosine**	0.047 [0.023 0.125]	0.064 [0.023 0.190]	0.604
**Fluconazole**	0.380 [0.125 0.750]	0.380 [0.190 0.625]	0.853
**Itraconazole**	0.094 [0.032 0.120]	0.064 [0.032 0.120]	0.585
**Posaconazole**	0.032 [0.023 0.094]	0.023 [0.016 0.078]	0.143
**Voriconazole**	0.016 [0.008 0.120]	0.012 [0.007 0.023]	0.405
**Caspofungin**	0.064 [0.032 0.125]	0.094 [0.079 0.190]	0.019
***C. parapsilosis(sl)***			
**Amphotericin B**	0.190 [0.079 0.500]	0.500 [0.410 1.000]	0.004
**Flucytosine**	0.047 [0.016 0.064]	0.077 [0.032 0.125]	0.134
**Fluconazole**	1.000 [0.410 1.875]	1.000 [0.750 2.000]	0.410
**Itraconazole**	0.109 [0.023 0.282]	0.125 [0.047 0.235]	0.892
**Posaconazole**	0.064 [0.025 0.218]	0.079 [0.051 0.173]	0.570
**Voriconazole**	0.064 [0.012 0.120]	0.032 [0.007 0.064]	0.170
**Caspofungin**	0.500 [0.250 1.500]	1.500 [0.190 2.000]	0.279

Data are presented as median [interquartile range]. MIC, Minimal Inhibitory Concentration.
